# Micromechanical Characterization of AlCu Films for MEMS Using Instrumented Indentation Method

**DOI:** 10.3390/ma17194891

**Published:** 2024-10-05

**Authors:** Dongyang Hou, Yuhang Ouyang, Zhen Zhou, Fang Dong, Sheng Liu

**Affiliations:** 1 The Institute of Technological Sciences, Wuhan University, Wuhan 430072, China; 2022106520011@whu.edu.cn (D.H.); 20222106520021@whu.edu.cn (Y.O.); zhen.z@whu.edu.cn (Z.Z.); 2 School of Power and Mechanical Engineering, Wuhan University, Wuhan 430072, China; 3 Key Laboratory for Hydropower Transients of Ministry of Education, Wuhan University, Wuhan 430072, China

**Keywords:** dimensional analysis, finite element simulation, micromechanical property, MEMS film, nanoindentation

## Abstract

The micromechanical properties (i.e., hardness, elastic modulus, and stress–strain curve) of AlCu films were determined by an instrumented indentation test in this work. For three AlCu films with different thicknesses (i.e., 1 µm, 1.5 µm, and 2 µm), the same critical ratio (*h*_max_/*t*) of 0.15 and relative indentation depth range of 0.15–0.5 existed, within which the elastic modulus (i.e., 59 GPa) and nanoindentation hardness (i.e., 0.75 GPa, 0.64 GPa and 0.63 GPa for 1 µm, 1.5 µm and 2 µm films) without pile-up and substrate influence can be determined. The yield strength (i.e., 0.754 GPa, 0.549 GPa and 0.471 GPa for 1 µm, 1.5 µm and 2 µm films) and hardening exponent (i.e., 0.073, 0.131 and 0.150 for 1 µm, 1.5 µm and 2 µm films) of Al-(4 wt.%)Cu films for MEMS were successfully reported for the first time using a nanoindentation reverse method. In dimensional analysis, the ideal representative strain *ε*_r_ was determined to be 0.038. The errors of residual depth *h*_r_ between the simulations and the nanoindentation experiments was less than 5% when the stress–strain curve obtained by the nanoindentation reverse method was used for simulation.

## 1. Introduction

Advanced functional devices (e.g., sensors [1], power devices [2] or storage [3], solar cells [4]) are composed of complex stacks of multiple layers of heterogeneous thin films (e.g., metals, semiconductors, and dielectric ceramics) [5], and each layer has the role of one or more functions (e.g., signal transmission [6], energy conversion [7], insulation [8]). The bond pads (i.e., metal thin films), as metalized areas in integrated circuits, are used to connect the circuit on the chip to probe needles for wafer testing or to the pins of the outside package for assembly [9]. The demand for shrinking the device dimension and package minimization has raised a serious concern about the mechanical robustness of the thin-film stacked structure with the bond pad [10], especially in the wafer probe testing and wire bonding process [11]. Moreover, the mechanical properties of thin films fundamentally affect the long-term functional reliability of micro–electro–mechanical systems (MEMS) devices [12]. Therefore, understanding the mechanical response of metal bond pad thin films is crucial for optimizing the processes associated with MEMS manufacturing.

Several techniques have been developed to evaluate the mechanical properties of thin films with micrometer thickness, such as bulge tests, tensile tests, substrate curvature, X-ray diffraction, and nanoindentation methods [13]. The bulge and tensile tests require removing the thin film from the substrate, which is difficult for many films. The substrate curvature, X-ray diffraction, and nanoindentation techniques can be used to determine the mechanical properties of films attached to substrates, but they are limited by the spatial resolution of the X-ray diffraction method [14] and nonuniformity in film stress or thickness for the substrate curvature method [15], respectively. Nanoindentation is a powerful tool for characterizing the material properties of thin films due to its high load and displacement resolution, high testing efficiency, and microdamage nature [16,17,18].

The empirical rule of 10% was first proposed by Bückle [19] to address the effect of the substrate, which is not always valid for all materials. Pelletier et al. [20] found that the critical ratio of indentation depth to the film thickness for extracting the mechanical properties of Al_2_O_3_ films is 0.35. Lim et al. [21] observed the pile-up behavior of residual nanoindentation imprints on Al films on sapphire substrates and suggested considering the projected contact area of the imprints when using nanoindentation technology to determine the hardness and elastic modulus of the film to improve the accuracy of measurement results. Gouldstone et al. [22] found that the nanoindentation response of Al and Cu films is mainly composed of pure elastic behavior with intermittent microplasticity, and the overall plastic response of the film is related to its thickness. Lee et al. [23] found that the pop-out event of Au films during the unloading curve is related to the amorphous transformation. Cao et al. [24] revealed the indentation size effects of Au and Ag films using the strain gradient theory and found that the variation in the yield strengths and hardnesses of the films with grain size was in accordance with the Hall–Petch relationship. Wang et al. [25] proposed a method for quantitatively evaluating the influence of the substrate on the measurement of the mechanical properties of a single-layer thin film by the loading curve exponent. Ma et al. [26] studied the effects of indentation depth and substrate on indentation hardness and proposed a simple method for predicting the intrinsic hardness of thin films.

The plastic characteristics of thin films can be directly extracted from nanoindentation experiments using dimensional analysis and finite element simulation methods [27,28]. Dao et al. [29] developed a reverse analysis procedure and expressions for predicting the plastic properties of materials based on the nanoindentation technique. Lee et al. [30] reduced data sensitivity and experimental errors, using loading curvature and the ratio of plastic work to total mechanical work as independent parameters of indentation response. Zhao et al. [31] proposed an inverse method for measuring the elastic modulus, yield stress, and hardening exponent of thin films using a sharp indentation test and validated on a Cu thin film/Si substrate system. In references [32,33], the researchers validated the effectiveness of parameterization in inversion analysis methods. In references [34,35], the researchers emphasize that the finite element method can help evaluate the elastic–plastic properties of thin films from nanoindentation experimental data. Zhao et al. measured the coefficient of thermal expansion, elastic modulus, and Poisson’s ratio of Al-(0.5 wt.%)Cu films deposited on Si (100) substrates using a wafer curvature technique. Read et al. [36] reported the yield strength, elastic modulus, and failure elongation of Al-(0.5 wt.%)Cu films deposited on Si substrates using tensile testing combined with digital image correlation methods at 25–150 °C. Jeon et al. [37] proposed a new specimen micro-fabrication process for evaluating the interfacial toughness of Al-(0.5 wt.%)Cu films on Si substrates, and verified the reliability of the proposed method based on the finite element method and linear elastic fracture mechanics theory. However, it remains a challenge to reverse the plastic characteristics of MEMS films that are only a few micrometers thick by nanoindentation tests, and the mechanical properties of AlCu films for MEMS have yet to be systematically reported.

The current work aimed to estimate the micromechanical properties (i.e., hardness, elastic modulus, and stress–strain curve) of AlCu films with different thicknesses by instrumented indentation tests. The microstructure of AlCu films was investigated by scanning electron microscopy, energy dispersive spectrometer, and atomic force microscopy. The hardness and elastic modulus of AlCu films with pile-up behavior were evaluated based on nanoindentation tests and residual imprints. The stress–strain curves of AlCu films were obtained by combining finite element simulation and dimensional analysis, and imported into ABAQUS Workbench for finite element simulation to verify the accuracy of plastic parameters. The result of this research can effectively facilitate the acquisition of true plastic parameters of metal bond pads for MEMS and is helpful in providing guidance for improving the wafer probe testing and optimizing the associated processes of MEMS manufacturing.

## 2. Materials and Methods

### 2.1. Microstructural and Mechanical Characterization

AlCu films with different thicknesses were deposited by magnetron sputtering from an Al-(4 wt.%)Cu target on the commercial Si (111) substrates. Before depositing, the Si substrate is soaked and cleaned in isopropanol and baked in a 120 °C oven for 1 h. The thin-film deposition was carried out at 160 °C in an Ar atmosphere with a vacuum degree of 5.0 × 10^−6^ Torr. The deposition process was divided into four stages, as shown in Table 1, and the thickness of the thin film depends on the deposition time. The prepared wafer was sawn mechanically into a 3 × 3 cm^2^ die size. Scanning electron microscopy (SEM, Tescan CLARA, Brno, Czech Republic) with an energy dispersive spectroscopy (EDS) was utilized to characterize the thickness and composition of AlCu films. The vertically placed AlCu film samples were embedded with resin material using a metallographic mounting machine (XQ-2B, Xiwaka Precision Measuring Instrument Co., Ltd., Dongguan, China), and then polished by the automatic mechanical grinding and polishing machine (MP-3S-2, Huayin Testing Instrument Co., Ltd., Zhengzhou, China) to obtain the polished sample section. The surface roughness of the samples was measured by atomic force microscopy (AFM, JPK NanoWizard, Bruker, Billerica, MA, USA), and then the surface roughness profile of the samples was obtained using Gwyddion 2.66 software.

Nanoindentation tests were performed using Anton Paar (Graz, Austria) nanoindentation tester NHT^2^ with a Berkovich indenter at room temperature and different maximum loads *F*_max_. The durations for loading and unloading were 30 s each, with a holding period of 10 s and a data acquisition frequency of 10 Hz [38,39]. The loading segment of the curve is written by Kick’s law [40]:

(1) $$F_{L}=Ch^{2}$$

where *F*_L_ is the loading load, *C* is the loading curvature, and *h* is the indentation depth.

According to the Oliver–Pharr (OP) method, reduced elastic modulus E* can be calculated [38]:

(2) $$E^{*}=\frac{S\sqrt{\pi }}{2\beta \sqrt{A_{p}}},\;E^{*}={\left[\frac{1-v^{2}}{E_{\mathrm{IT}}}+\frac{1-\nu_{i}^{2}}{E_{i}}\right]}^{-1}$$

where *S* = *dF*/*dh*, representing the slope of the initial unloading curve, is the contact stiffness, *β* (=1.034 for Berkovich indenter [41]) is a numerical factor related to the shape of the indenter, and the projected contact area *A*_p_ is a function of contact depth *h*_c_ and can be obtained by calibrating on a reference material with known elastic modulus and Poisson’s ratio [42]; *E*_IT_ and *v* are the elastic modulus and Poisson’s ratio of the specimen, respectively; *E*_i_ = 1141 GPa and *ν*_i_ = 0.07 are the elastic modulus and Poisson’s ratio of the diamond indenter used in the present study. The Poisson’s ratio *v* of AlCu films is 0.35 [43].

Nanoindentation hardness *H*_IT_ can be calculated as follows:

(3) $$H_{\mathrm{IT}}=\frac{F_{\max}}{A_{p}}$$

The ratio of the load-over-stiffness square can be expressed as [41]:

(4) $$\frac{H_{\mathrm{IT}}}{{\left(E^{*}\right)}^{2}}=\frac{4\beta^{2}F_{\max}}{\pi S^{2}}$$

### 2.2. Dimensional Analysis

The stress-strain relationship of the materials with an assumption of isotropic hardening can be expressed as [30]:

(5) $$\sigma =\left\{ \begin{array}{l} E\varepsilon ,\;\sigma \leq \sigma_{y}\\ \sigma_{y}{\left(1+\frac{E}{\sigma_{y}}\varepsilon_{p}\right)}^{n},\sigma \geq \sigma_{y} \end{array}\right.$$

where *σ*_y_ is the yielding strength, *ε*_p_ is the plastic strain, and *n* is the hardening exponent.

Assuming the Berkovich indenter is rigid, the loading load *F*_L_ is a function of the mechanical parameters of materials, and the indentation imprint parameters and can be expressed as [44]:

(6) $$F_{L}=F_{L}\left(h,E,\nu ,E_{i},\nu_{i},\sigma_{r},n\right)=F_{L}\left(h,E^{*},\sigma_{r},n\right)$$

where *σ*_r_ is the representative stress.

Based on the dimensional analysis, Equation (6) becomes:

(7) $$\frac{C}{\sigma_{r}}=\prod_{1}\left(\frac{E^{*}}{\sigma_{r}},n\right)$$

Similarly, the unloading load *F*_U_ can be expressed as:

(8) $$F_{U}=F_{U}\left(h,h_{\max},E^{*},\sigma_{r},n\right)$$

When *F*_U_ = 0, the specimen is fully unloaded, and *h* = *h*_r_. Therefore, Equation (8) becomes:

(9) $$\frac{h_{r}}{h_{\max}}=\prod_{2}\left(\frac{E^{*}}{\sigma_{r}},n\right)$$

where *h*_r_ is the residual depth, and *h*_max_ is the maximum indentation depth.

### 2.3. Finite Element Simulation

**Figure 1:**
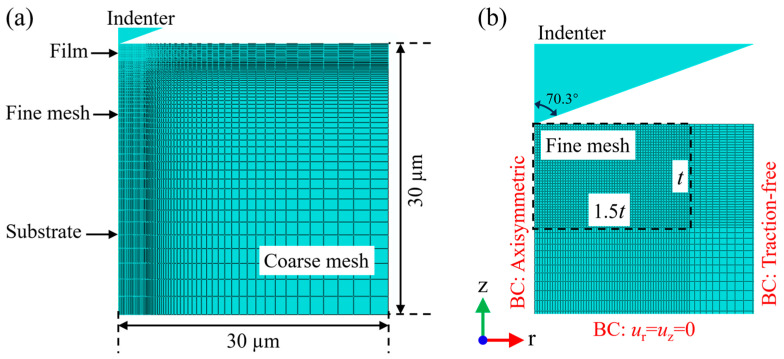
Schematic illustration of the geometry and mesh of 2D axisymmetric model used for nanoindentation simulations: (**a**) global view and (**b**) local view.

Figure 1a shows the geometry and mesh of a two-dimensional (2D) axisymmetric nanoindentation model built by the commercial software ABAQUS 2022. The FE simulation in this study was implemented based on the static equilibrium equation inside ABAQUS/CAE, and an incremental–iterative method based on the Newton–Raphson method was adopted to solve the non-behavior equations. A 2D model was used instead of a 3D model to enhance computational efficiency, since the simulation results of the 2D model have been proven to be consistent with those of the 3D model [45]. An asymmetrical conical indenter with a half-angle of 70.3° was built to be equivalent to the Berkovich indenter, which can be modeled as a rigid body due to the elastic modulus (1141 GPa) of the indenter being far greater than that of AlCu films. The surface-to-surface contact type was used, and the contact between the indenter and the film was assumed to be frictionless, as the influence of friction on the *F*-*h* curves can be neglected [46].

**Figure A1:**
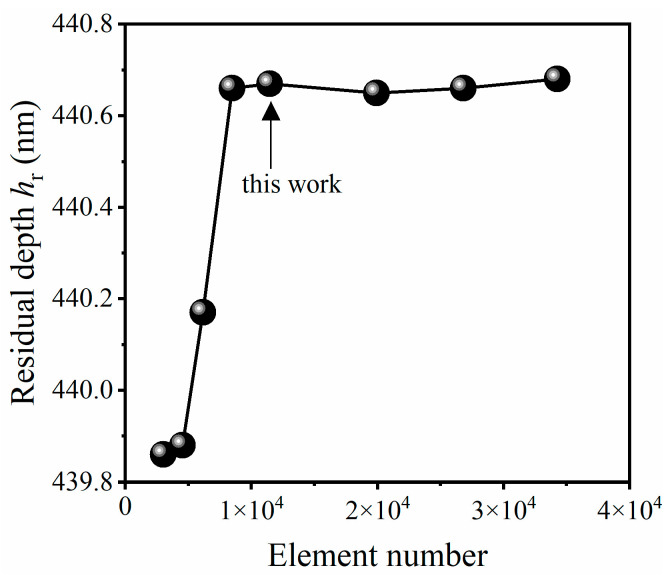
The independence test results of FEM mesh.

Figure 1b displays the local view of the 2D model. The meshes were refined to an element size of 50 nm in the region beneath the indenter tip to ensure numerical convergence and the independence of mesh size, see Figure A1 in Appendix B, and became gradually coarser as the distance from the tip increased. As for the boundary conditions of the film/substrate system, the element node at the plane of the symmetry axis was constrained horizontally; the right boundary is a free surface due to it being sufficiently far away from the indentation location; and the element node at the bottom of the model was completely fixed for all the degrees-of-freedom. The indenter was restricted to vertical movement, and the load type was displacement control. The *F*-*h* curve was obtained based on the reaction force and displacement of a reference point on the rigid indenter [47]. The frequency of data points is increased using the time-points option to obtain a more accurate unloading curve, with each point spaced 0.5–1 nm for different indentation depths.

Assuming all interfaces have perfect adhesion, without considering the intrinsic stresses within the stacked structure caused by manufacturing processes, the film and the substrate materials are isotropic [48,49]. The 2D nanoindentation finite element model (FEM) of the AlCu/Si system was built using a mesh type of the linear quadrilateral CAX4R with reduced integration. For each sample, 60 sets of FEM simulations considering plastic parameters were performed to obtain stress–strain curves, respectively: *σ*_y_ varied from 0.05 to 5 GPa and *n* from 0 to 0.5. The initial values of *σ*_y_ and *n* are set to 0.05 and 0, respectively.

### 2.4. Framework of Reverse Analysis

**Figure 2:**
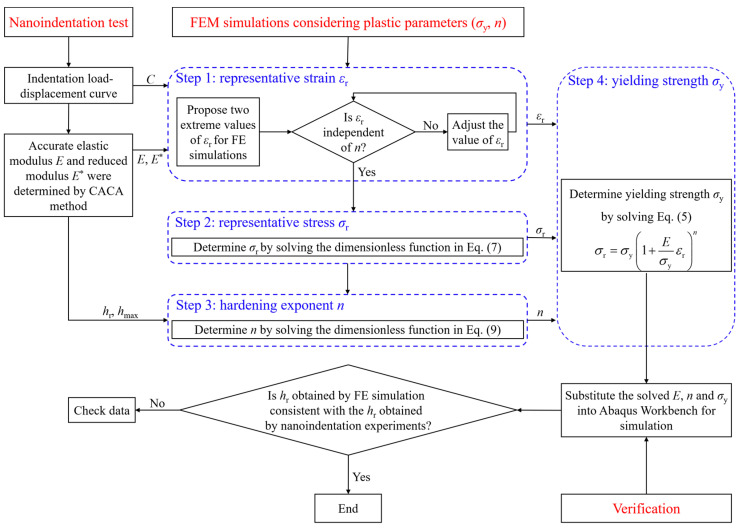
Flow chart of the proposed reverse analysis method.

The framework of the proposed reverse analysis method in this study was summarized in Figure 2. The reverse analysis procedure can be implemented through the following program: the representative strain *ε*_r_ independent of the hardening index *n* is first determined by assuming two extreme values of *ε*_r_; the representative stress *σ*_r_ is determined by solving the dimensional function in Equation (7) after choosing *ε*_r_; and then *n* is determined by solving the dimensional function in Equation (9); the yield strength *σ*_y_ was determined by substituting *E*, *ε*_r_, *σ*_r_, and *n* into Equation (5), and then obtaining the complete stress–strain curve; finally, the accuracy of the reverse method was verified by substituting the stress–strain curve into the ABAQUS Workbench.

## 3. Results

### 3.1. Microstructure Characterization

**Figure 3:**
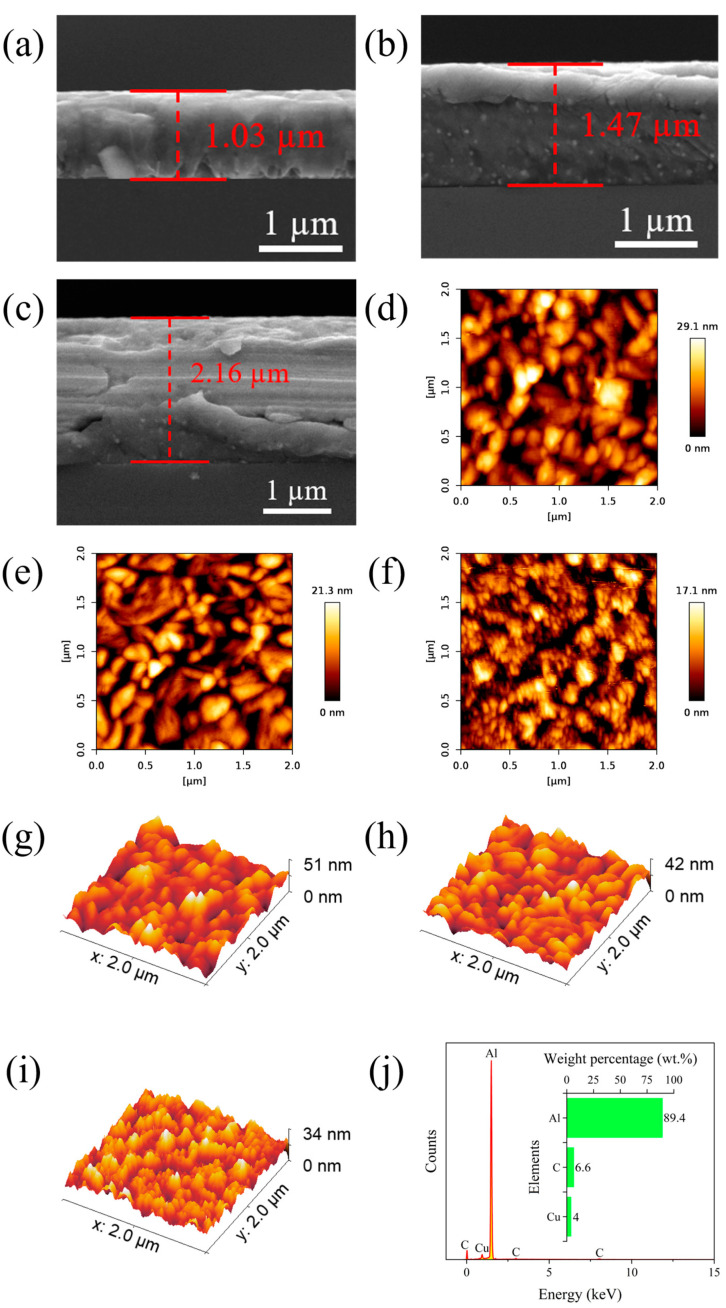
The microstructural characterization of AlCu films with different thicknesses: SEM images of the cross-section for (**a**) 1 µm, (**b**) 1.5 µm and (**c**) 2 µm films; AFM images of surface topography for (**d**) 1 µm, (**e**) 1.5 µm and (**f**) 2 µm films; the roughness profile of surface topography for (**g**) 1 µm, (**h**) 1.5 µm and (**i**) 2 µm films; and (**j**) EDS spectra.

Figure 3a–c shows the SEM images of cross-sections of AlCu films with different thicknesses, indicating that the film thickness *t* of the three samples are approximately 1 μm, 1.5 µm, and 2 µm, respectively. Figure 3d–f and Figure 3g–i show AFM images and the roughness profile of the surface topography of AlCu films with different thicknesses, respectively. The root-mean-square (RMS) surface roughness, which is one of the most common parameters for characterizing the surface morphology [50] of AlCu films, was in the order of a nanometer (i.e., 6.6 nm, 4.8 nm and 3.9 nm for *t* = 1 µm, 1.5 µm and 2 µm films, respectively) over a 2 × 2 μm^2^ area, indicating the good smoothness of the films, ensuring the accuracy and consistency of nanoindentation experimental results. Figure 3j shows the EDS spectra of AlCu films, and corresponding peaks of Al, Cu, and C with no impurity peaks were observed. Al and Cu contents in AlCu films are approximately 89.4 wt.% and 4 wt.%, respectively, which is consistent with the composition of the target used for depositing thin films.

### 3.2. Elastic Modulus and Hardness Characterization

**Figure 4:**
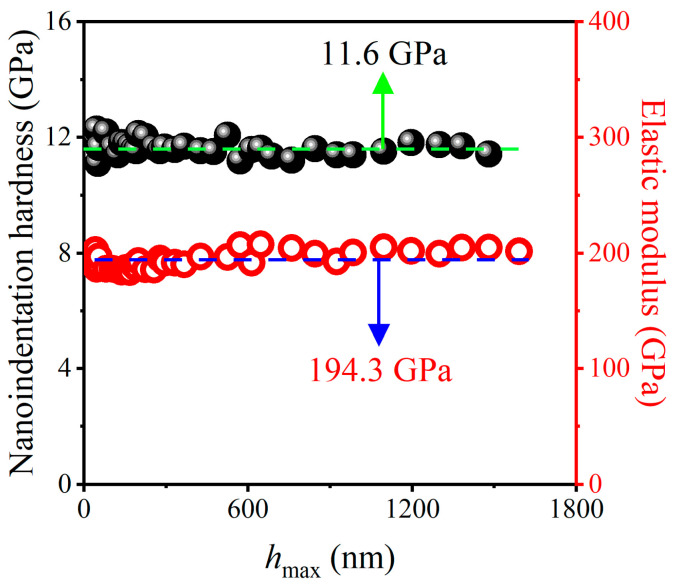
Variation in *H*_IT_ and *E*_IT_ of Si (111) substrate with *h*_max_.

Nanoindentation tests were also performed on Si substrates, which can provide the baseline data for the AlCu/Si system. Figure 4 shows the variation in nanoindentation hardness *H*_IT_ and elastic modulus *E*_IT_ of the Si (111) substrate with the maximum indentation depth *h*_max_, and the Poisson ratios of 0.18 were used for Si (111) [51]. The *H*_IT_ and *E*_IT_ of Si (111) substrate exhibited an approximately constant value between the maximum indentation depth *h*_max_ of 0 and 1800 nm. The average nanoindentation hardness value of 11.6 GPa was obtained, which is close to the 11.8 GPa reported in the literature [52]. The average elastic modulus value of 194.3 GPa was determined, which is almost the same as the 193 GPa obtained by nanoindentation tests [53].

**Figure 5:**
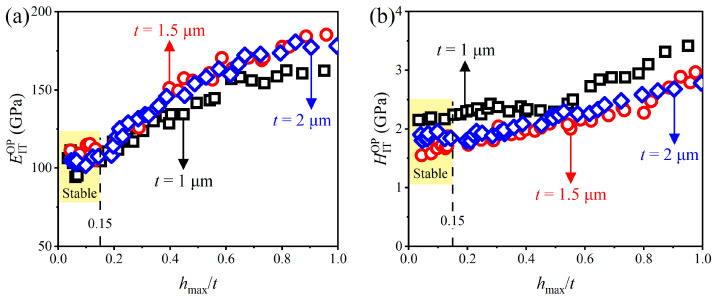
Variations in (**a**) $E_{\mathrm{IT}}^{\mathrm{OP}}$ and (**b**) $H_{IT}^{OP}$ obtained by the OP method with *h*_max_/*t*.

**Figure 6:**
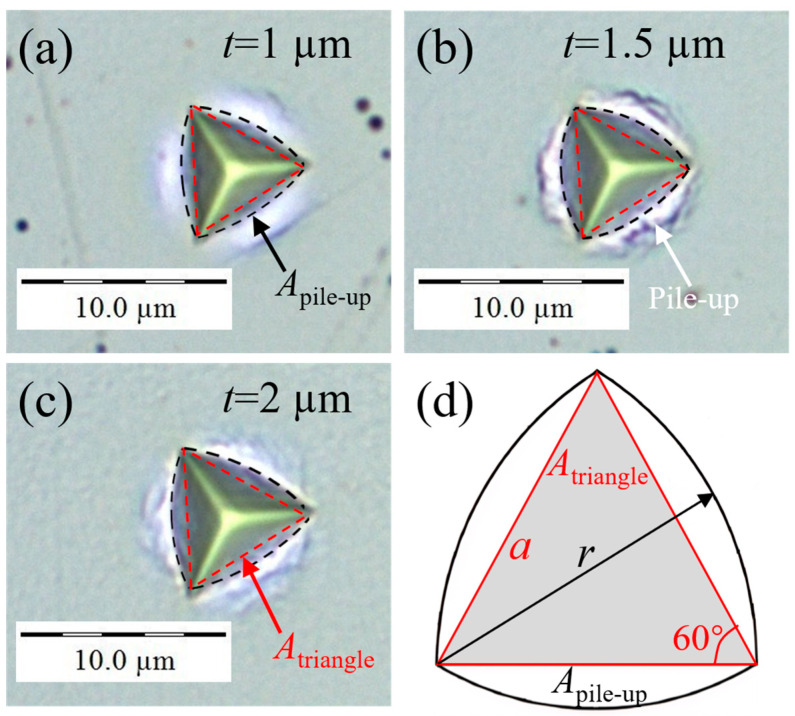
Optical images of residual imprints of AlCu films with different thicknesses under applied load of 50 mN: (**a**) 1 µm, (**b**) 1.5 µm, and (**c**) 2 µm; (**d**) schematic illustration for calculating the projected area considering pile-up.

Figure 5 shows the variations in elastic modulus $E_{\mathrm{IT}}^{\mathrm{OP}}$ and indentation hardness $H_{\mathrm{IT}}^{\mathrm{OP}}$ calculated by the OP method (i.e., Equations (2) and (3)) with relative indentation depths *h*_max_/*t*. Under small *h*_max_/*t* (<0.15), $E_{\mathrm{IT}}^{\mathrm{OP}}$ and $H_{\mathrm{IT}}^{\mathrm{OP}}$ exhibit a stable platform, while the average values of $E_{\mathrm{IT}}^{\mathrm{OP}}$ (i.e., 99.8 GPa, 110.4 GPa and 101.5 GPa for *t* = 1 µm, 1.5 µm and 2 µm films, respectively) and $H_{\mathrm{IT}}^{\mathrm{OP}}$ (i.e., 2.18 GPa, 1.70 GPa and 1.87 GPa for *t* = 1 µm, 1.5 µm and 2 µm films, respectively) within the stable platform are significantly larger than the reasonable ranges of the similar films (e.g., for elastic modulus, Al-(0.5 wt.%) Cu films are 40–59 GPa [36,37,54], Al films are 23–64 GPa [55,56,57,58]; for hardness, Al-(0.5 wt.%) Cu films are 0.17–0.25 GPa [59], Al films is 0.4–0.8 GPa for [60]), which is attributed to the OP method, which does not consider the effect of pile-up behavior, see Figure 6, on evaluated material properties [61]. Under large *h*_max_/*t* (>0.15), $E_{\mathrm{IT}}^{\mathrm{OP}}$ and $H_{\mathrm{IT}}^{\mathrm{OP}}$ increase with increasing *h*_max_/*t*, which can be explained by the substrate effects [62].

Figure 6a–c shows the optical images of residual imprints of AlCu films with different thicknesses under an applied load of 50 mN. An obvious pile-up was observed, and the pile-up material formed an arc around the edges of the indent, indicating that the calculating actual contact area (CACA) methods can be used to correct the pile-up errors in the OP analysis [30,63]. When pile-up occurs, the total areas *A*_t_ supporting the load are composed of the projected contact area *A*_p_ calculated by the OP method and the areas *A*_pile-up_ produced by pile-up, as follows:

(10) $$A_{t}=A_{p}+A_{\mathrm{pile}\text{-}\mathrm{up}},A_{p}\approx A_{\mathrm{triangle}}=\frac{\sqrt{3}}{4}a^{2},\;a=2\sqrt{3}\tan\theta h_{c}$$

where *A*_triangle_ and *a* are the area and edge length of the Berkovich imprint in Figure 6d, respectively, which was assumed to be equilateral triangular; *θ* = 65.27° is the centerline-to-face angle of the Berkovich indenter [41].

Assuming that the center of the arc is located at the opposite corner of the imprint, then the radius *r* of the arc is approximately equal to *a*, and *A*_pile-up_ can be expressed as:

(11) $$A_{\mathrm{pile}\text{-}\mathrm{up}}=3\left(\frac{\pi r^{2}}{6}-\frac{\sqrt{3}}{4}a^{2}\right)\approx \left(\frac{2\pi -3\sqrt{3}}{4}\right)a^{2}$$

**Figure 7:**
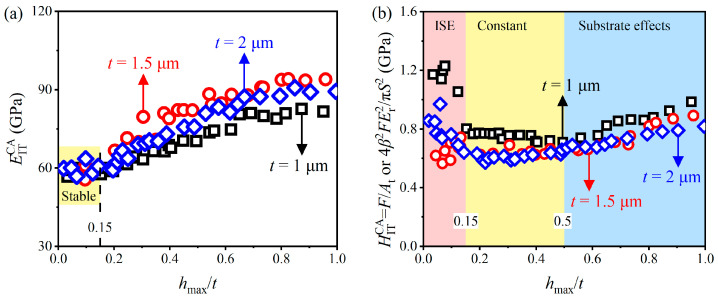
Variations in (**a**) $E_{\mathrm{IT}}^{\mathrm{CA}}$ and (**b**) $H_{\mathrm{IT}}^{\mathrm{CA}}$ obtained by the CACA method with *h*_max_/*t*.

Figure 7 shows the variations in elastic modulus $E_{\mathrm{IT}}^{\mathrm{CA}}$ and indentation hardness $H_{\mathrm{IT}}^{\mathrm{CA}}$ calculated by the CACA method (i.e., Equations (10) and (11)) with relative indentation depths *h*_max_/*t*, and the $E_{\mathrm{IT}}^{\mathrm{CA}}$ and $H_{\mathrm{IT}}^{\mathrm{CA}}$ of three AlCu thin films with different thicknesses show the same trend with *h*_max_/*t*. Under small *h*_max_/*t* (<0.15), $E_{\mathrm{IT}}^{\mathrm{CA}}$ can be approximated to be constant (i.e., $E_{\mathrm{IT}}^{\mathrm{CA}}$ = 58.9 GPa, 58.3 GPa and 59.4 for *t* = 1 µm, 1.5 µm and 2 µm films, respectively), which lies within the reasonable range. As anticipated, the elastic response of the films is independent of thickness [22]. Under large *h*_max_/*t* (>0.15), $E_{\mathrm{IT}}^{\mathrm{CA}}$ increases with increasing *h*_max_/*t* due to the substrate effects [64], since the observed rise follows the elastic modulus of the substrates. Under small *h*_max_/*t* (<0.15), $H_{\mathrm{IT}}^{\mathrm{CA}}$ decreases with *h*_max_/*t* due to the indentation size effect [65]; the constant values of $H_{\mathrm{IT}}^{\mathrm{CA}}$ (i.e., *H*CA IT = 0.75 GPa, 0.64 GPa and 0.63 GPa for *t* = 1 µm, 1.5 µm and 2 µm films, respectively) can be approximated for intermediate indentation depths (i.e., 0.15 < *h*_max_/*t* < 0.5), and can be regarded as the ones without the influence of the substrate; $H_{\mathrm{IT}}^{\mathrm{CA}}$ increases with *h*_max_/*t* due to the substrate effects under large *h*_max_/*t*. The results indicate that the nanoindentation hardness values of soft films on hard substrates at intermediate indentation depths (i.e., 0.15 < *h*_max_/*t* < 0.5 for AlCu films) can be accurately obtained by correcting the error of the elastic modulus caused by pile-up behavior, which is to be expected, as the plastic field under the indenter is a short-range field compared to the elastic field [20,64].

### 3.3. Stress–Strain Characteristic

**Figure 8:**
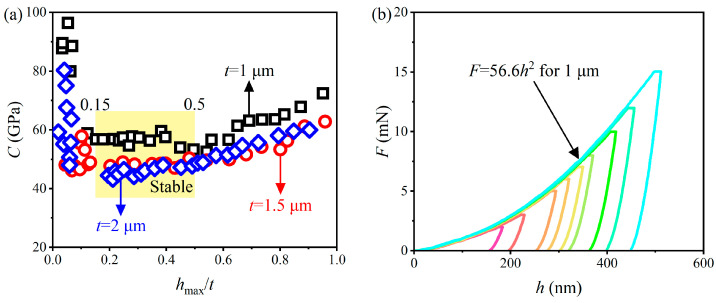

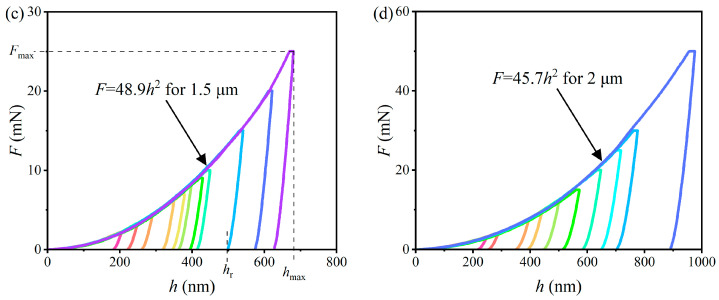
(**a**) Variations in *C* with *h*_max_/*t*; *F*-*h* curves of AlCu films with different thicknesses under different *F*_max_: (**b**) 1 µm, (**c**) 1.5 µm, and (**d**) 2 µm.

The maximum indentation depth is maintained within the AlCu film thickness of 15–50% to avoid the poor fit of the curvature *C* data by Kick’s law under small *h*_max_/*t* (<0.15) and the effect of Si substrate under large *h*_max_/*t* (>0.5), as shown in Figure 8d. Figure 8b–d shows the indentation load *F*-displacement *h* curves of three AlCu films at different *F*_max_, and the hysteresis phenomenon between the loading and unloading curves indicates that the AlCu film undergoes elastic–plastic deformation during nanoindentation. The smooth loading and unloading curves indicate no cracks or fractures in the AlCu film during nanoindentation [66,67]. The loading curves under different loads follow the same trace, indicating the good repeatability of the nanoindentation experiment. The loading curves of thin films with different thicknesses reflect the ability of the films to resist external deformation. Under the same load, it can be seen that *h*_max_ of 1 μm AlCu film is the smallest, *h*_max_ of 1.5 μm AlCu film is in the middle, and *h*_max_ of 2 μm AlCu film is the largest, indicating that the ability of AlCu films to resist external deformation decreases with increasing *t*. The average value of *C* decreases with an increase in the film thickness *t* by 0.15 < *h*_max_/*t* < 0.5, and the average value *C* of three AlCu films is listed in Table 2.

**Figure 9:**
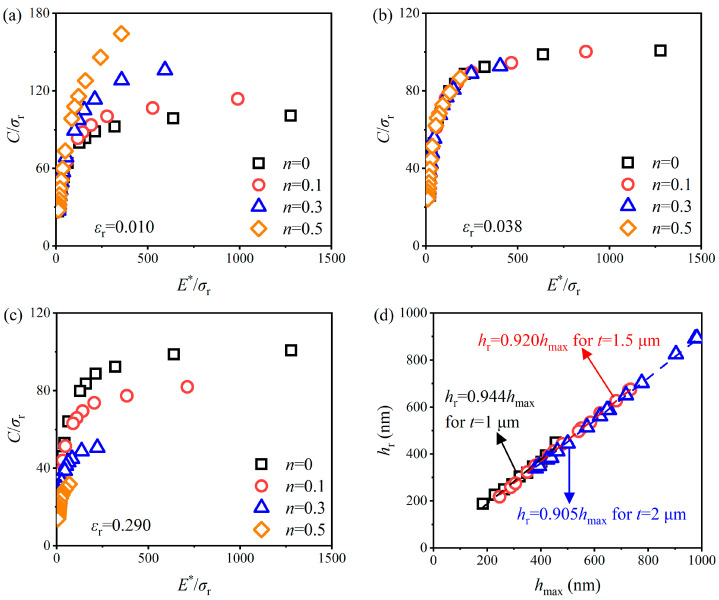
Variation in *C*/*σ*_r_ with E*/*σ*_r_ for 2 µm AlCu film at different *n* and *ε*_r_: (**a**) *ε*_r_ = 0.010, (**b**) *ε*_r_ = 0.038, and (**c**) *ε*_r_ = 0.290; and (**d**) the relationships between *h*_r_ and *h*_max_ for 0.15 < *h*_max_/*t* < 0.5.

Figure 9a–c shows the variation in *C*/*σ*_r_ (i.e., ∏_1_ function) with E*/*σ*_r_ for 2 µm AlCu film at different hardening exponents *n* and representative strain *ε*_r_. Within the range of E*/*σ*_r_ from 10.61 to 1278.3, *C*/*σ*_r_ increased as *n* increased when *ε*_r_ < 0.038; when *ε*_r_ > 0.038, *C*/*σ*_r_ decreased with an increase in *n*; *C*/*σ*_r_ was independent of *n* when *ε*_r_ = 0.038, indicating that power law plastic responses of all stress–strain curves can exhibit the same stress *σ*_r_ at a plastic strain of 3.8%, which is close to the representative strain value (i.e., 0.033) reported by Dao [29], for given E* and *C*. Figure 9d shows the proportional relationship between residual depth *h*_r_ and the maximum indentation depth *h*_max_. The large *h*_r_/*h*_max_ (i.e., 0.905–0.944) corroborated elastic–plastic and pile-up behavior for AlCu films [68]. The value of *ε*_r_ and *h*_r_/*h*_max_ will serve as a critical reference for determining the plastic characteristics of AlCu films for MEMS.

By choosing *ε*_r_ = 0.038, Equation (7) can be expressed as:

(12) $$\frac{C}{\sigma_{r}}=\Pi_{1}\left(\frac{E^{*}}{\sigma_{r}}\right)$$

**Figure 10:**
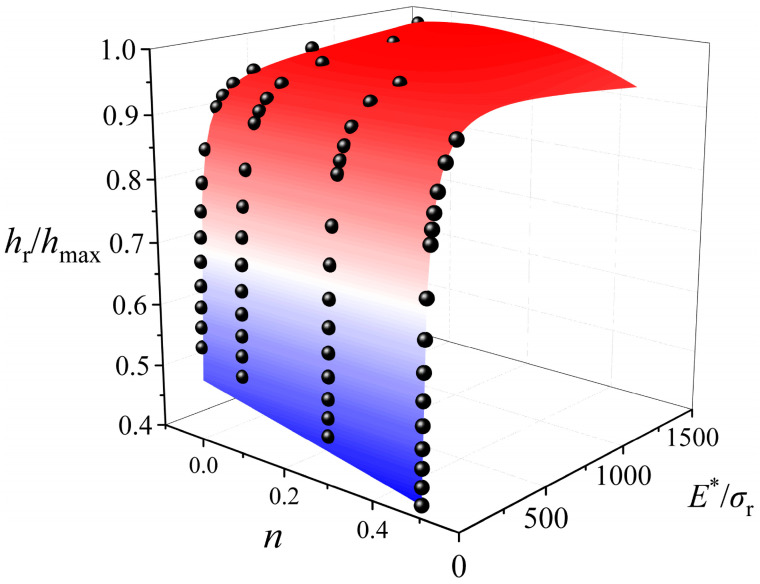
Variation in *h*_r_/*h*_max_ as a function of E*/*σ*_r_ and *n* for 2 µm AlCu film.

The dimensionless function ∏_1_ can be established by fitting all data points presented in Figure 9b, and then the value of *σ*_r_ corresponding to a plastic strain of 3.8% can be calculated by substituting the nanoindentation test data *C* and *E*^∗^ into ∏_1_. The dimensionless function ∏_2_ can be determined by fitting all data points presented in Figure 10. The ∏_1_ and ∏_2_ of three AlCu films with different thicknesses are the same, due to the difference in the elastic modulus between them being small [44]. The ∏_1_ and ∏_2_ in the Appendix A can be well-fitted, since their formats refer to Dao’s study [29] and accurate *ε*_r_ values can be determined for AlCu films. The value of *n* can be calculated by substituting *h*_r_/*h*_max_ and E* obtained by the nanoindentation tests, and *σ*_r_ calculated by ∏_1_ into ∏_2_. Finally, *σ*_y_ can be solved by substituting *σ*_r_, *n*, *ε*_r_ into Equation (5).

**Figure 11:**
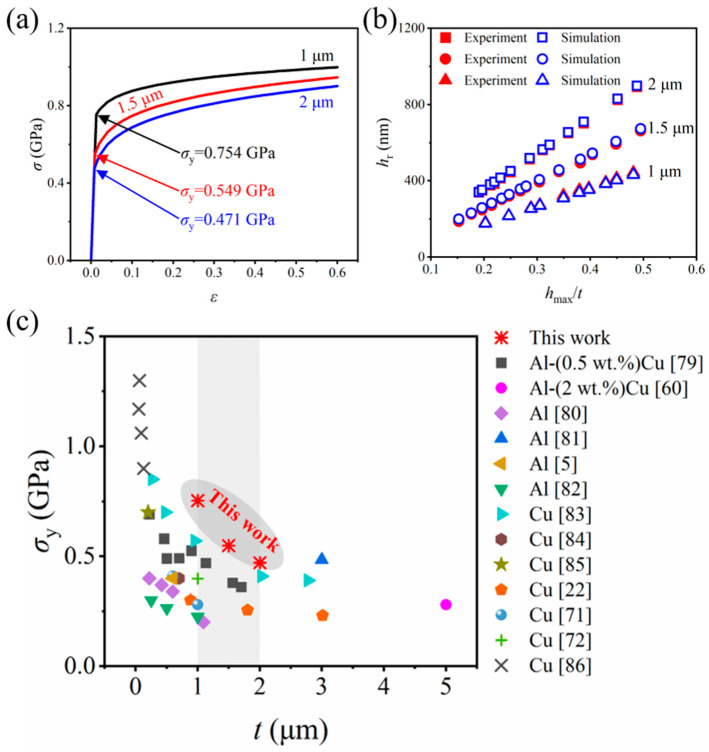
The results and validation of *σ*_y_ obtained by the nanoindentation reverse method for Al-(4 wt.%)Cu films with different thicknesses: (**a**) stress–strain curves; (**b**) the comparison of *h*_r_ between simulation and experimental results; (**c**) the comparison of *σ*_y_ between this work and that reported by various methods in the literature: Macionczyk [79], Nix [60], Doerner [80], Cai [81], Yeo [5], Stone [82], Yu [83], Hommel [84], Balk [85], Gouldstone [22], Keller [71], Flinn [72], and Zhang [86].

Figure 11a shows the stress–strain curves of three AlCu films with different thicknesses, and the material parameters for calculating stress–strain curves of AlCu films are listed in Table 2. The yield strength *σ*_y_ decreases with increasing film thickness *t*, which is consistent with the inference made from the curvature and X-ray diffraction measurements of thin films [69,70,71,72]. The 2 μm AlCu film will reach *σ*_y_ more readily, resulting in irrecoverable plastic deformation, which is consistent with the results of *F*-*h* curves from the nanoindentation tests. The hardening exponent *n* increases with increasing *t*, indicating that the “hardening” is more significant during the plastic deformation stage [73]. The stress–strain curves of AlCu films with different thicknesses in Figure 11a were imported into ABAQUS Workbench for finite element simulations, and the results are shown in Figure 11b. The residual depth *h*_r_ between simulations and nanoindentation tests are in good agreement, and the errors all remained within 5%, indicating that the plastic parameters of AlCu films in this work are pretty accurate. Figure 11c shows the comparison of *σ*_y_ between this work and that reported by various methods in the literature, and *σ*_y_ decreases with increasing *t*. The *σ*_y_ reversed by nanoindentation tests in this work is slightly larger than that reported by the literature at the same as *t*, which can be attributed to such factors as the different experimental methods [22], indentation size effect [74], the differences between the localized and macroscopic deformation [75], the influence of grain size [76], residual stress [77], temperature [78], and the fact that the non-ideal indenter shape is not considered in the FEM [43]. Therefore, the *σ*_y_ curves of the AlCu films obtained in this work are reasonable considering the complexity of influencing factors.

Currently, the accuracy of the proposed method is significantly dependent on the measurement accuracy of residual depth, which can be improved by constructing dimensionless functions based on the energy theory. In addition, the proposed method is limited by the constitutive model, which is a common limitation of the indentation reversed method, since power-law equations may not describe many stress–strain curves well. In the future, the automatic acquisition of material parameters with the combination of nanoindentation and finite element simulation methods based on algorithm optimization, especially an artificial intelligence technique, will be further developed, which can greatly improve the reverse efficiency of material parameters and help break through the limitations of power–law equations.

## 4. Conclusions

The microstructure and surface topography of three AlCu films with different thicknesses were characterized by SEM, EDS, AFM and XRD. We established an analytical procedure of measuring the micromechanical properties (i.e., hardness, elastic modulus, and stress–strain curve) of Al-(4 wt.%)Cu films for MEMS by nanoindentation tests. The conclusions are as follows:

(1) For AlCu films with a thickness of several micrometers, nanoindentation measurement errors caused by pile-up behavior can be corrected by determining the actual contact area.
(2) The nanoindentation hardness of soft films on hard substrates can be accurately obtained by correcting the error of elastic modulus caused by pile-up behavior.
(3) The elastic modulus of Al-(4 wt.%)Cu films is independent of film thickness and is approximately 59 GPa. The nanoindentation hardnesses of Al-(4 wt.%)Cu films for 1 µm, 1.5 µm and 2 µm were 0.75 GPa, 0.64 GPa and 0.63 GPa, respectively.
(4) With the increasing film thickness, the yield strength of Al-(4 wt.%)Cu films decreased, and the hardening exponent increased.

## Figures and Tables

**Table 1:** The summary parameters of AlCu films deposited by magnetron sputtering, which were provided by a commercial semiconductor manufacturing company.

Stage	Base Pressure (Pa)	Sputtering Power (kW)	Gas Flow (sccm)
I	1	5	120
II	1	0.2	120
III	1	0.2	120
IV	54	10	140

**Table 2:** Summary parameters for calculating stress–strain curves of AlCu films.

*t* (µm)	*E* (GPa)	E* (GPa)	*C* (GPa)	*σ*_r_ (GPa)	*h*_r_/*h*_max_	*n*	*σ*_y_ (GPa)
1	58.9	63.4	56.6	0.834	0.944	0.073	0.754
1.5	58.3	62.8	48.9	0.679	0.920	0.131	0.549
2	59.4	63.9	45.7	0.613	0.905	0.150	0.471

## Data Availability

The original contributions presented in the study are included in the article, further inquiries can be directed to the corresponding authors.

## Appendix A

The ∏_1_ and ∏_2_ functions used in this work are detailed as follows:

$$\begin{array}{l} \frac{C}{\sigma_{r}}=\prod_{1}\left(\frac{E^{*}}{\sigma_{r}}\right)\\ =-0.6998{\left[\mathrm{Ln}(\frac{E^{*}}{\sigma_{r}})\right]}^{3}+7.231{\left[\mathrm{Ln}(\frac{E^{*}}{\sigma_{r}})\right]}^{2}\\ -1.435\mathrm{Ln}(\frac{E^{*}}{\sigma_{r}})-4.727 \end{array}$$

$$\begin{array}{l} \frac{h_{r}}{h_{\max}}=\prod_{2}\left(\frac{E^{*}}{\sigma_{r}}\right)\\ =(-0.1261n^{2}+0.0165n+0.0060){\left[\mathrm{Ln}(\frac{E^{*}}{\sigma_{r}})\right]}^{3}\\ +(0.1996n^{2}+0.0025n-0.1147){\left[\mathrm{Ln}(\frac{E^{*}}{\sigma_{r}})\right]}^{2}\\ +(-0.8084n^{2}+0.0425n+0.7556)[\mathrm{Ln}(\frac{E^{*}}{\sigma_{r}})]\\ +(0.9970n^{2}-0.2629n-0.7281) \end{array}$$

## Appendix B

Figure A1 shows the independence test results of FEM mesh in this study. When the element number of FEM is larger than 8466, the residual depth *h*_r_ obtained by nanoindentation ABAQUS simulation is independent of the element number. The total element number of FEM used in this study is 11,462, ensuring the accuracy of the simulation results.
